# Lost in translation: confusion on resection and dissection planes hampers the interpretation of pathology reports for perihilar cholangiocarcinoma

**DOI:** 10.1007/s00428-019-02621-w

**Published:** 2019-08-24

**Authors:** Eva Roos, Lotte C. Franken, Eline C. Soer, Jeanin E. van Hooft, R. Bart Takkenberg, Heinz-Josef Klümpen, Johanna W. Wilmink, Marc J. van de Vijver, Thomas M. van Gulik, Joanne Verheij

**Affiliations:** 1 Department of Surgery, Cancer Center Amsterdam, Amsterdam UMC, University of Amsterdam, Meibergdreef 9, 1105 AZ Amsterdam, The Netherlands; 2 Department of Pathology, Cancer Center Amsterdam, Amsterdam UMC, University of Amsterdam, PO Box 22660, Mijbergdreef 9, 1105 AZ Amsterdam, The Netherlands; 3 Department of Gastroenterology and Hepatology, Tytgat Institute, Amsterdam UMC, University of Amsterdam, Meibergdreef 9, 1105 AZ Amsterdam, The Netherlands; 4 Department of Medical Oncology, Cancer Center Amsterdam, Amsterdam UMC, University of Amsterdam, Meibergdreef 9, 1105 AZ Amsterdam, The Netherlands

**Keywords:** Perihilar cholangiocarcinoma, Residual disease status, Periductal dissection plane, Pathology report

## Abstract

In perihilar cholangiocarcinoma (PHC), interpretation of the resection specimen is challenging for pathologists and clinicians alike. Thorough and correct reporting is necessary for reliable interpretation of residual disease status. The aim of this study is to assess completeness of PHC pathology reports in a single center and assess what hampers interpretation of pathology reports by clinicians. Pathology reports of patients resected for PHC at a single expert tertiary center drafted between 2000 and 2018 were assessed. Reports were assessed regarding completeness, according to the guideline of the International Collaboration on Cancer Reporting (ICCR). A total of 146 reports were assessed. Prognostic tumor characteristics such as vasoinvasive growth and perineural growth were missing in 30/146 (34%) and 22/146 (15%), respectively. One or more planes were missing in 94/146 (64%) of the reports, with the periductal dissection plane missing in 51/145 (35%). Residual disease could be re-classified from R0 to R1 in 22 patients (15%). Reasons for R1 in these patients were the presence of a positive periductal dissection plane (*n* = 2), < 1-mm margin at the periductal dissection plane (*n* = 11), or liver parenchyma (*n* = 9). Completeness of reports improved significantly when drafted by an expert HPB pathologist. This study demonstrates that pathology reporting of PHC is challenging. Reports are frequently incomplete and often do not incorporate assessment of all resection planes and the dissection plane. The periductal dissection plane is frequently overlooked, but is a major cause of residual disease.

## Introduction

Perihilar cholangiocarcinoma (PHC) is a rare adenocarcinoma that arises from the biliary tract epithelium in the hilum of the liver [1–3]. Tumors are classified as PHC when they originate between the second bifurcation of the hepatic duct and proximal of the cystic duct. Curative treatment is only feasible for minority of patients [4]. The preferred surgical therapy is an (extended) hemihepatectomy, with resection of the extrahepatic biliary tract in combination with complete lymphadenectomy of the hepatoduodenal ligament [5–7]. Median overall survival was 40 months in resected patients, provided that complete resection is achieved [8]. Surgical margin status affects disease-free and overall survival. However, some studies show that the current definition of residual disease in PHC is insufficient due to poor assessment of resection margins [9]. Patients with PHC have a high risk of recurrent disease and standard adjuvant treatment is lacking [10].

Ambiguity on the correct way to report on resection margins and to determine residual disease status impedes correct interpretation of pathology data, correct risk assessment, and the consistent design of future studies [11–16]. Adequate staging and assessment of radicality of the tumor are contingent on careful pathological assessment and reporting [11, 17]. However, the best way to assess resection margins to determine residual disease (R0/R1) remains a subject of debate in PHC [18]. R0 is described as microscopically negative surgical margins by the College of American Pathologists. This means that even though the tumor may reach closer than 1 mm to the margin, there is R0 disease if it does not extent into the resection plane [19]. On the other hand, the British Royal College of Pathologists defines R1 as the presence of tumor cells within 1-mm margin of the resection plane.

In 2018, a consensus guideline was published by the International Collaboration on Cancer Reporting (ICCR), containing the essential parameters to be incorporated in the pathology report for cholangiocarcinoma [20]. It states that in PHC, R0 means a tumor-free margin of ≥ 1 mm, as distance in millimeters between the tumor and resection or dissection plane is prognostic for survival [21–23]. However, many authors have shown that reporting on margin status and standard parameters is frequently incomplete [20, 24–27].

**Fig. 1:**
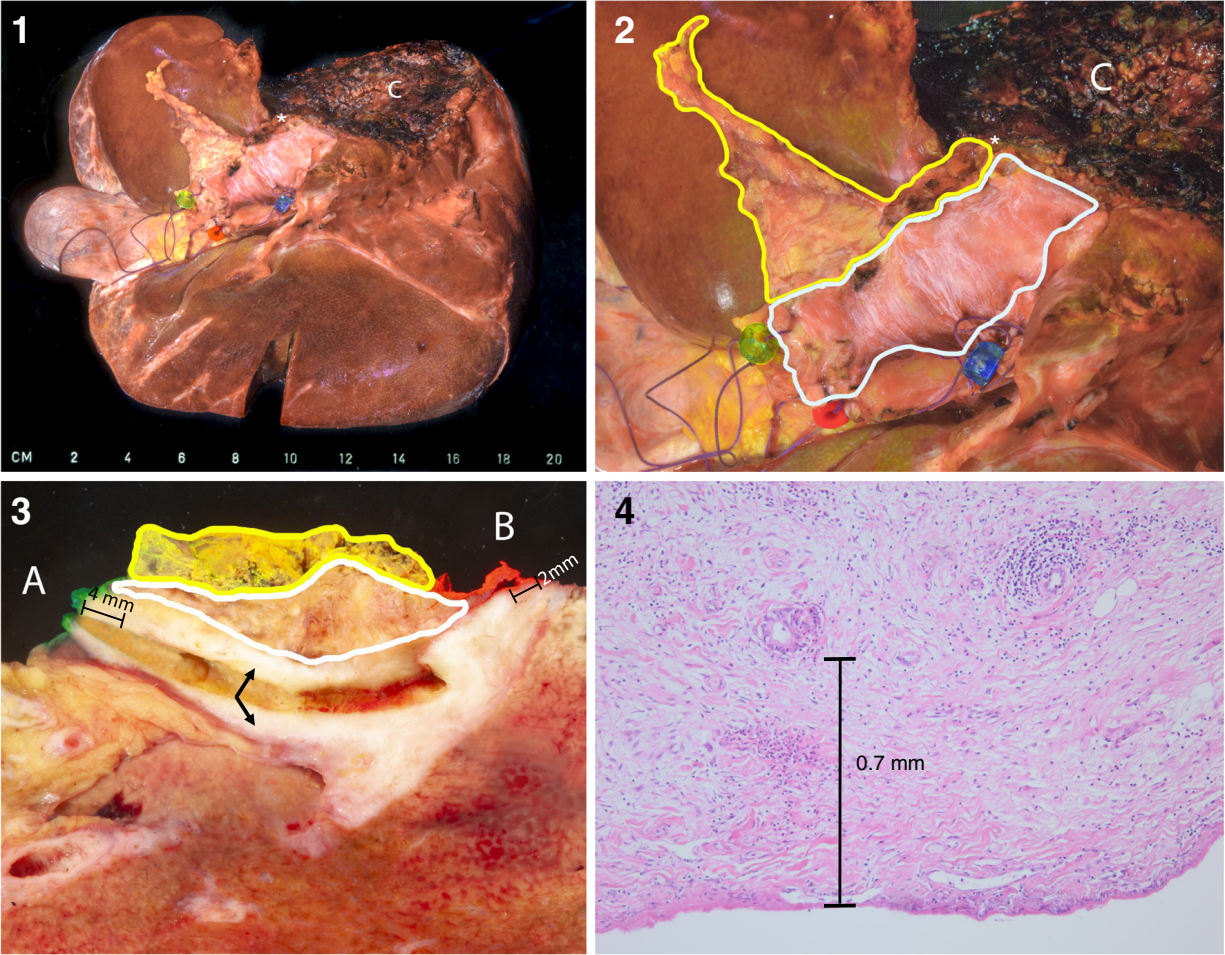
(1) Overview of surgical specimen in extended right hemihepatectomy (incl segment 1) with extrahepatic bile duct resection. (2) Close-up hilar area. The fine fibers of the smooth peritoneal surface can be appreciated (outlined in white), as well as the slightly irregular periductal dissection plane (outlined in yellow). (3) The annular resection margins of the common bile duct (A) and left hepatic duct (B) were sampled, and the underlying tissue was colored in inked green and red, respectively. The specimen was cut along the bile duct after probing. A part of the bile duct shows a white, fibrotic, and thickened wall due to tumor involvement (arrows), with a 4-mm clearance from the common bile duct margin and 2-mm clearance from the left hepatic duct. (4**)** Microscopic slide showing a tumor gland in relation to the peritoneal surface. Periductal dissection plane was not involved in this specimen, as shown in (3). (1–3) *Green bead* common bile duct, *blue bead* portal vein, *red bead* hepatic artery, *white asterisk* left hepatic duct, *yellow line* periductal dissection plane, *white line* peritoneal surface; (C) liver parenchyma

A second, major problem is the complexity of the surgical specimen and its relevant resection and dissection planes. Treatment most often is an (extended) hemihepatectomy with external bile duct excision. In this case, there are five resection planes—common bile duct, segmental biliary branches, hepatic artery, portal vein, liver parenchyma—and one periductal dissection plane (see Fig. 1). Although the authors have chosen to refer to this dissection margin as periductal dissection plane, others have reported this as (circumferential) dissection margin [22] or periductal soft tissue circumferential margin [21]. The periductal dissection plane consists of a circumferential surgical dissection plane, opposed to the peritoneal surface on the other side (see Fig. 1). This dissection plane is of importance in the assessment of margin status, since tumors which invade the hilar soft tissue and grow within 1 mm of the soft tissue dissection surface should be classified as R1 [23]. Understanding and reporting of these six planes are crucial to adequately determine residual disease status. It is often unclear in reports which ones have been assessed [21]. The importance to report on these planes separately has been repeatedly emphasized [21].

In this article, we studied the quality and completeness of reporting in terms of resection planes and other standard pathological parameters (e.g., tumor size, vasoinvasive growth, perineural growth). We assess the value by drafting under supervision of a dedicated gastrointestinal/hepatopancreaticobiliary pathologist.

## Materials and methods

In this retrospective cohort study, we evaluated pathology reports of patients with confirmed perihilar cholangiocarcinomas that were resected in a tertiary referral center in The Netherlands between 2000 and 2018. Patients with intraductal papillary neoplasms of the bile duct, intrahepatic cholangiocarcinoma invading the hilum, benign disease, or any other malignancy were excluded. The need for ethical approval was waived by the Medical Ethics Review Committee of the Amsterdam UMC (W18_235).

### Data assessment

Data were retrospectively retrieved from the original pathology reports. Pathology reports were assessed by two independent investigators (ER, LCF). When consensus was not met, decision was made by an expert hepatopancreatobiliary (HPB) pathologist (JV). Reports were assessed regarding completeness of the following clinicopathological parameters: type of resection, orientation, gross features, liver- and tumor-derived microscopic features, frozen sections, resection planes, and lymph nodes (see Table 1). Items were assessed for their reporting in the full text and/or in the conclusion of the pathology report. In 2008, our department adopted a workflow in which only dedicated pathologists with expertise in gastrointestinal pathology assessed the specimens and drafted the reports. It was recorded if pathology reports were drafted by an expert HPB pathologist (after 2008) or a general pathologist (before 2008). For all annular resection planes (CBD, hepatic ducts, hepatic artery, and portal vein), margins were assessed as positive or negative, since they are assessed using annular grossing technique. Because these slices generally are 1 to 3 mm, a “negative” annular plane ensures a margin of > 1 mm. An annular segment of the most proximal (hepatic duct) and distal (CBD) resection planes is generally sent in during surgery for assessment by frozen section. If this frozen section is tumor-negative, this ensures a negative margin. If a margin in millimeters was provided, this was recorded as stated in the report. For non-annular planes (periductal and liver parenchyma), margins were assessed as < 1 mm or ≥ 1 mm, as determined by the ICCR guideline. If millimeters were not provided, resection planes were recorded as positive or negative as stated in the report.

**Table 1:** Pathology parameters that were assessed

Type of resection	(extended) Hemihepatectomy or external bile duct resection, since resection planes are not identical in these two treatments
Surgical specimen’s orientation marks	As provided by the surgeon: provided or not provided. Orientation marks are usually not provided for the liver parenchyma and periductal dissection plane
The tumor’s gross features	Size in mm
The liver parenchyma’s microscopic features	Inflammation, fibrosis, steatosis, (secondary) sclerosing cholangitis due to obstruction
Tumor’s microscopic features	Histological subtype, differentiation grade, and perineural growth. Vasoinvasive growth was subdivided in major vessel involvement or microscopic vessel involvement. Major vessel involvement was determined as invasion of the lumen of the portal vein and/or hepatic artery. Involvement of only the media was determined as negative involvement, since the biological implications of vessel involvement are mainly dependent on intraluminal tumor growth
Lymph nodes	Total amount of derived lymph nodes and ratio of positive lymph nodes
Frozen sections	Frozen sections of the proximal and distal bile duct resection plane, hepatic artery, portal vein, lymph nodes, and other biopsies or lesions It was noted whether frozen sections were concordant or discordant with the final histological diagnosis
Surgical specimen’s resection planes	Common bile duct, segmental branches, portal vein and hepatic artery, liver parenchyma, periductal dissection margin
Residual disease based on resection margins	Positive frozen section, positive resection plane in the surgical specimen, or a resection plane with a margin of < 1 mm

As mentioned, tumor-negative slides of the annular plane margins (CBD, hepatic duct, portal vein, hepatic artery) ensure a margin of > 1 mm (due to the annular grossing technique). However, this is not the case for the liver parenchyma and periductal dissection plane margins, as with negative margins, tumor cells could technically still be within < 1 mm of the margin. Therefore, specimen slides of patients that were assessed as “true R0” based on their pathology report (all margins described and none of them positive) were reassessed for the liver parenchyma margin and/or periductal dissection plane if millimeters were not provided. Also, in case of the description in a pathology report of tumor cells *infiltrating closely to* but not *into* the concerning resection plane, this specific margin was reassessed in the specimen slice.

### Interpretation of residual disease

Patients with complete description of all resection planes and the dissection plane (CBD, hepatic ducts, hepatic artery, portal vein, liver parenchyma, periductal dissection plane) and without any positive resection or dissection plane, were considered R0. Residual disease was defined as a positive frozen section or a positive resection plane in the surgical specimen. Patients with missing resection or dissection plane and no other “positive” resection planes were considered “R unclear.” R-status as documented at the postoperative multidisciplinary meeting was recorded. Discrepancies between residual disease based on the pathology reports and defined at the postoperative multidisciplinary meeting were listed.

### Statistical analysis

Data were analyzed using descriptive statistics. Analyses were performed using SPSS 24 software.

## Results

A total of 146 patients undergoing resection for PHC between 2000 and January 2018 were included in this study. There were 132 patients undergoing liver resection with five relevant resection planes (distal bile duct, proximal duct, portal vein, hepatic arty, liver parenchyma) and one periductal dissection plane, 14 patients underwent extrahepatic bile duct resection without liver resection with three relevant planes (distal, proximal, and periductal dissection planes), and one patient underwent liver resection without extrahepatic bile duct resection with two relevant resection planes (liver parenchyma and hepatic duct). In two patients, additional pancreatoduodenectomy was performed due to a positive frozen section at the distal resection margin (CBD). Patient characteristics are listed in Table 2.

**Table 2:** Characteristics of patients undergoing resection for PHC. ^#^Interquartile range (IQR), ^$^standard deviation (SD), number (*n*)

Patient characteristics	*n* = 146
Age^$^	63 (10)
Female, *n* (%)	54 (37)
Bismuth-Corlette, n (%)	
I	4 (3)
II	14 (10)
IIIa	68 (47)
IIIb	32 (22)
IV	28 (19)
Size in mm^#^	28 (20–40)
Liver resection	132 (91)
Left	52 (40)
Extended left	7 (5)
Right	30 (23)
Extended right	42 (32)
Minor	2 (2)
Pancreatoduodenectomy	3 (2)
Portal vein reconstruction	39 (27)

### Completeness of reported parameters

Marking of the planes by bead or suture was provided by the surgeon in 89% (130/146) of all surgical specimens (see Table 3). The periductal dissection plane was never marked by a bead or suture. Tumor size was described in 82% (120/146) of all reports. Differentiation grade of the tumor was described in 88% of all reports (39/146). Perineural growth and vasoinvasive growth were described in 85% (124/146) and 66% (96/146) of the reports, and present in 77% (113/124) and 43% (41/96), respectively.

**Table 3:** Reported parameters in the pathology report and results of pathology assessment. Common bile duct (CBD). ^#^IQR. Absolute numbers, percentages between bars (%)

	Mentioned in text	Result
Markings provided	130/146 (89)	
Tumor size^#^	120/146 (82)	28 (20–40)
Lymph node status^#^	143/146 (98)	0 (0–1) positive
Microscopic features of tumor		
Perineural growth	124/146 (85)	113/124 (77)
Vasoinvasive growth	96/146 (66)	41/96 (43)
Differentiation	139/146 (95)	
Poor		22 (16)
Poor-moderate		11 (8)
Moderate		66 (47)
Moderate-well		6 (5)
Well		22 (16)
Microscopic features of liver parenchyma	121/132 (91)	
Distal margin (CBD, *n* = 145)		
Frozen section	144/145 (99)	11 positive (5 negative at re-resection)
Histology in case of lacking frozen section	1/145	1 negative
Missing	0	
Orientation provided by surgeon	118	
Proximal margin (hepatic duct, *n* = 145)		
Frozen section	131/145 (90)	24 positive (9 negative at re-resection)
Histology in case of lacking frozen section	13/145 (9)	4 positive, 8 negative, 1 < 1 mm
Missing	2/145 (1)	
Orientation provided by surgeon	125	
Portal vein resection plane (*n* = 132)		
Frozen section	26/132 (20)	7 positive
Histology in case of lacking frozen section	83/132 (63)	11 positive, 63 negative, 5 < 1 mm, 4 > 1 mm
Missing	25/132 (20)	18/25 orientation was not provided
Orientation provided by surgeon	102	
Hepatic artery resection plane (*n* = 132)		
Frozen section	17/132 (11)	2 positive (1 negative at re-resection)
Histology in case of lacking frozen section	57/132 (43)	2 positive, 52 negative, 1 < 1 mm, 2 > 1 mm
Missing	60/132 (45)	55/60 orientation was not provided
Orientation provided by surgeon	60	
Liver parenchyma resection plane (*n* = 132)		
Histology	105/132 (80)	8 positive, 52 negative, 12 < 1 mm. 32 > 1 mm
Missing	27/132 (20)	
Periductal dissection plane(*n* = 145)		
Histology dissection plane	93/145 (64)	10 positive, 21 negative, 43 < 1 mm, 19 > 1 mm
Missing	51/145 (35)	

### Completeness of reporting on frozen sections and resection margins

In all cases, at least one frozen section of a resection plane was performed. The distal (CBD) and proximal (hepatic) resection planes were assessed by either frozen section or histology of the plane in 100% (145/145) and 99% (144/145) of cases, respectively (see Table 3). However, description of the hepatic artery and periductal dissection plane was missing in 45% (60/132) and 35% (41/145), respectively (see Table 3).

### Interpretation by expert pathologists

Reports were more frequently complete when they were drafted by a dedicated gastrointestinal pathologist (see Table 4); the number of reports that incorporated all relevant resection planes increased from 12 to 45% before and after 2008 (*p* < 0.001), and there was a significant improvement in the reporting vasoinvasive growth, perineural growth, differentiation grade, and description of the periductal dissection plane (see Table 4).

**Table 4:** Evolution over time of assessment by pathologists. Absolute numbers, percentages between bars (%)

Missing variables	Before 2008 (*n* = 41)	After 2008 (*n* = 105)	*p* value
Marking of planes provided by surgeon missing	8/41 (20)	8/105 (8)	*0.039*
Vasoinvasive growth missing	23/41 (56)	27/105 (25)	*0.001*
Perineural growth missing	14/41 (34)	8/105 (8)	*< 0.001*
Differentiation missing	3/41 (7)	13/105 (13)	0.657
Amount of missing planes			
Complete	5/41 (12)	47/105 (45)	*< 0.001*
1 missing	13/41 (31)	29/105 (28)	0.624
2 missing	10/41 (24)	21/105 (20)	0.560
3 missing	9/41 (22)	8/105 (8)	*0.015*
4 missing	3/41 (7)	0	*0.005*
5 missing	1/41 (2)	0	0.108
Periductal dissection plane missing	28/41 (68)	25/105 (24)	*< 0.001*
Residual disease unclear	16/41 (35)	30/105 (29)	0.222

### Revisions of margins in specimen slides

Revisions of tumor slides took place in 23 cases: in 11 potential “true” R0 patients (where the margin at the liver parenchyma and/or periductal plane was described as “negative”) and in 12 patients due to a description in the pathology report of a tumor *infiltrating closely to* but not *into* the concerning resection plane. Of these 11 potential “true” R0 patients, revisions resulted in a margin of < 1 mm at the periductal dissection plane in 5/11 patients. Revisions of the specimen slides of the 12 patients with a description of a tumor *infiltrating closely to* but not *into* the concerning resection plane (2 liver parenchyma and 10 periductal dissection plane) resulted in a margin of < 1 mm in 10/12 patients and of > 1 mm in 2/12 patients.

### Interpretation of residual disease

By re-evaluating the pathology reports by the investigators, we could re-classify the following 26 cases based on the new ICCR guidelines. In the pathology report of 12 R0 patients, the periductal dissection plane was described as positive (*n* = 2) or with a margin of < 1 mm (*n* = 10). In 9 R0 patients, the margin at the liver parenchyma resection plane was described as < 1 mm. There were 5 patients in whom the periductal dissection plane was described as “negative,” but when revising the specimen slides, tumor reached within 1 mm of the dissection plane. Although these 26 patients were, at the time, documented as R0, based on the ICCR guidelines, these patients would be classified as R1. Additionally, there were 4 reports that were wrongly interpreted as R0 during the multidisciplinary meeting, although a positive resection plane was described. Last, in patients without any positive resection or dissection plane, missing planes in the original report might have led to a false interpretation of R0 status (R unclear). This was the case in 41/146 (28%) patients (assessed as R0 at a multidisciplinary meeting, although residual disease strictly could not be determined due to missing resection planes).

## Discussion

The drafting of a pathology report of a resection specimen of PHC is notoriously difficult. Completeness of reporting on PHC varies in literature. In comparison with the national cohort of Chatelain et al. (*n* = 22 hospitals), in our single-center tertiary expert, both microscopic tumor features and resection planes were more frequently described than in the multicenter French study (differentiation was assessed in 70% of cases in the series by Chatelain et al. vs. 95% of cases in our series, perineural growth in 54% vs. 77%, angioinvasive growth in 33% vs. 43%, and the periductal dissection plane in 10% vs. 65%, respectively). However, there is still much room for improvement. In our cohort, incompleteness was most frequently caused by the lacking description of all six relevant resection and dissection planes. The hepatic artery plane and periductal dissection plane were missing in 45% and 35% of cases, respectively. Accordingly, marked margins of the hepatic artery as provided by the surgeon were most often absent and might have contributed to the high percentage of missing descriptions of this plane. Margins in millimeters were, even after the publication of the ICCR guidelines in 2012, often missing. There were 26 patients that were documented as R0 at follow-up, but could be re-classified as R1 according to the ICCR guidelines. Important tumor characteristics such as perineural and vasoinvasive growth are often only mentioned when positive, suggesting that characteristics are not described when absent. Quality of reports and completeness increased when drafted by dedicated GI pathologists.

In the assessment of residual disease, it is of importance that margins from dissection planes are assessed as well, preferably in millimeters. According to the ICCR guideline, a margin < 1 mm is R1. This also applies to the periductal dissection plane. The impact of circumferential dissection margins, similar to periductal dissection plane, on determination of residual disease is supported by its relevance in other gastrointestinal carcinomas. The circumferential dissection margin is of prognostic significance in pancreatic carcinoma and esophageal cancer [17, 28, 29]. It seems this plane is frequently overlooked because it is a dissection plane rather than a resection plane. This might lead to the limited value of residual disease status in PHC [9, 10, 30]. Therefore, it is of importance that the definition of the periductal dissection plane is clear.

In our study, we encountered several limitations when assessing the pathology reports. First, in many reports, not all margins are mentioned and if incorporated in the report, frequently exact number of mm is missing. It is often stated in a report that “all resection planes are free of tumor.” However, when not all planes are separately reported and no description of relevant resection planes is provided, we could not assume that all relevant resection planes were assessed. Furthermore, when margins are stated as being negative, margin distance may still have been less than 1 mm. This implies that R0 status in these cases is therefore uncertain. This underlines the importance of a correct future assessment of the specimen in the report in terms of margins and millimeters. The more objective the information, including absolute millimeters, the easier retrospective research will be, even after changing definitions of R status. In four reports that were documented as R0, there was a positive plane described. The reasons for the misinterpretation of residual disease could not be retrieved. In some cases, the relevant information was incorporated in the microscopic description, but not in the conclusion. This might have added to misinterpretation, as the microscopic description may be overlooked by clinicians.

There are several recommendations for both pathologists and clinicians. First, pathologists need to be aware that in a PHC resection specimen, in most cases, there are six resection and dissection planes to be reported. It is of importance to provide distance from tumor to margin in millimeters with respect to all resection planes, with special attention to the periductal dissection plane. Involvement of dedicated (HPB) pathologists improves correct reporting, even in expert centers. For surgeons, it is important to provide marks for relevant resection planes, ensuring that these will be recognized and properly assessed. Presence of the attending surgeon and the pathologist who assessed the specimen at the multidisciplinary meeting or a one-on-one “recap” of the assessment might be of great value to the interpretation of the report.

In conclusion, this study demonstrates that pathology reporting of PHC is challenging. Reports are frequently incomplete and often do not incorporate assessment of all relevant resection and dissection planes. Furthermore, tumor distance to the margin is frequently not reported. Incomplete reporting often leads to misinterpretation by clinicians. The periductal dissection plane is frequently overlooked, but is a major cause of residual disease. Quality of reporting in PHC benefits from supervision by an expert HPB pathologist. Agreement and awareness among all specialists involved in PHC concerning the relevant PA variables, and their definition (R status and distance 1 mm) is needed. Standardized reporting may be of aid in the complete documentation of relevant pathologic findings.
